# An online multilingual numeral dataset on Devnagari and English languages for pattern recognition research

**DOI:** 10.1016/j.dib.2023.109743

**Published:** 2023-10-31

**Authors:** Meenal K. Jabde, Chandrashekhar H. Patil, Amol D. Vibhute, Shankar Mali

**Affiliations:** a School of Computer Science, Dr. Vishwanath Karad MIT World Peace University, Pune, MH, India.; b Symbiosis Institute of Computer Studies and Research (SICSR), Symbiosis International (Deemed University), Pune-411016, MH, India.

**Keywords:** Air-written, Real-Time, Numeral recognition, Multilingual dataset, Devnagari and English datasets, Pattern recognition

## Abstract

The real-time air-writing multilingual datasets are widely used for several purposes, such as handwriting character or numeral pattern recognition. The air-writing systems are commonly used in operation theatres, online education systems, banking sectors, reservation counters, etc. However, the air-written numeral datasets are less for Devanagari and English languages needed for detecting patterns. Therefore, the present article introduces novel datasets written in the air for Devanagari and English. In addition, this article proposes a systematic novel strategy to collect the air-written multilingual numeral dataset from 100 individuals ranging in 20-40 age groups. The Devanagari and English 0-9 digits were ten times written in the air by every individual resulting in 10,000 images for each language. Thus, 20,000 images were generated and stored in the databases. The proposed dataset is freely available and could be a good resource for pattern recognition research.

Specifications Table

**Table:** 

Subject	Pattern recognition
Specific subject area	Computer vision methods for the recognition of online handwriting written in English and Devanagari languages
Type of data	Image
How the data were acquired	We have designed a GUI in Python to collect the dataset using a standard video camera for English and Devanagari numerals. A technique using the generic camera with a single writer and an uncontrolled environment is used to develop a dataset of numeral recognition. Each dataset has 10,000 air-written numbers collected from 100 users aged 20 to 40. Each of them wrote a given number 10 times in total. Thus, a user had written the 100 numerals of Devanagari and 100 numerals of English. An individual must write 0 to 9 digits in Devanagari and English script/language for a valid numeral. We have developed 20,000 numeral image datasets for Devanagari and English script/language.
Data format	Raw
Description of data collection	The fingertip position is provided using segmentation, fingertip recognition, trajectory approximation, and picture capture. The dataset is then updated with the numerical labels for each generated image occurrence. The users, such as post-graduate students, working professionals, and teachers/professors, are equally distributed as male and female. Thus, we have developed a real-time air-written numeral image dataset for Devanagari and English script/language.
Data source location	• Institution: School of Computer Science, MIT World Peace University. • City/Town/Region: Pune, Maharashtra. • Country: India
Data accessibility	Repository name: data.mendeley.com Data identification number: 10.17632/scnxzhgj83.1 Direct URL to data: https://data.mendeley.com/datasets/scnxzhgj83/1

## 1 Value of the Data

• The proposed datasets can be used in several research applications, such as covid-19 like pandemic situations, gesture-based interaction, augmented reality and virtual reality.
• The developed dataset helps handwriting detection and recognition of the multilingual numerical characters written in the air.
• The dataset plays a vital role in operation theatre or online education systems where pen-and-paper-based systems are not feasible or available. Thus, doctors and teachers can directly write in the air. Air-writing may be used to build virtual whiteboards where instructors can write, draw, or comment in the air, which is collected and presented on the students' screens in real-time. This enables more dynamic and compelling presentations, particularly in areas such as mathematics and physics, where number plays an important role.
• Air writing could be used to interact with augmented reality visualizations during surgery. Surgeons might use this to access and manipulate real-time number data included in pathology reports while performing procedures.
• Similarly, in the banking system, the air-written system can be used to write in the air to secure the systems directly. Customers with physical limitations who find standard typing difficult may find air-writing a more accessible communication method with financial services.
• Additionally, on the reservation counters of railways or flits, these systems can be used to directly book the tickets by writing the number in the air [1]. Customers might utilize air-writing to pick their date and ticket type instead of physically engaging with a ticketing machine or touch screen, avoiding the necessity for interaction with shared surfaces.
• The dataset can also develop machine or deep learning-based models to detect, recognize, and analyze handwriting characters and make decisions accordingly [2].
• Furthermore, suppose someone wants to work on a pattern recognition problem utilizing air-writing motions. In that case, they may need to construct their dataset by gathering data from volunteers or air-writing participants. As we acquired the data without using any external device or sensor, it offers a wide range of applications in pattern recognition research. The dataset has 10,000 images of each language, which is beneficial for many machine learning and deep learning techniques that require large datasets for training and testing.

## 2 Objective

No common air-written multilingual numeral dataset for English and Devnagari languages is needed for handwriting pattern recognition [2]. Therefore, the standard common dataset is required to develop novel models or algorithms that are designed for getting real-time output. Thus, the present article proposes the standard datasets containing more than 20,000 numeral images for Devanagari and English script/languages. To the best of our understanding, this is the most extensive dataset for the Devanagari and English script/language, and it can potentially become a go-to source for real-time handwriting pattern detection. In addition, we provide the framework to work on real-time air-written multilingual numeral recognition. The primary objectives are:

• To review the earlier standard online datasets.
• To develop the standard Devanagari and English script/languages datasets.
• To provide the framework to create a real-time air-written multilingual numeral dataset.
• To provide the possible applications, challenges, and research opportunities for generated standard datasets.

## 3 Data Description

### 3.1 Preprocessing and Saving the Numeral

The multilingual numeral dataset of air-writing videos of 2 different languages (Devanagari and English Script/language) using fingertip trajectories is developed in this article. The air writing videos are recorded on a web camera with backgrounds of varying brightness and complexity levels. The dataset includes samples for 0 to 9 digits in each language and recorded 1000 samples per language. One hundred participants wrote the Devanagari and English 0–9 numbers. The 100 participants range in age from 20 to 40 years old, consisting of 52 females and 48 men. The 100 participants must write each numeral (0 to 9) ten times in the air, resulting in ten thousand images for each language. As a result, 20, 000 images were created and saved in databases. A set of points for every trajectory includes fingertip position (x, y) and time t. Our dataset contains 20,000 samples, including 2000 samples per digit or 10,000 samples per language [3].

### 3.2 Final Datasets

The developed multilingual numeral datasets sample images for Devanagari and English numerals are provided in Table 1 with their features. The datasets are uploaded on https://data.mendeley.com/datasets/scnxzhgj83/1 for research purposes.

**Table 1:** Air writing multilingual numeral dataset samples in Devanagari and English numerals.

The influence of an air-written dataset for enhancing handwritten character or number pattern recognition systems depends on the dataset's quality, variety, and size and research and development activities in this specific domain of air-writing.

**Improved Generalization:** A well-constructed air-written dataset with various air-written letters or numerals can improve the pattern recognition system's generalization capacity. By including multiple writing techniques, speeds, and hand sizes, the system better responds to real-world settings where users may write differently in the air.

**Robustness to Noise:** Because of hand tremors or imperfect tracking, air-writing movements may introduce noise and fluctuations. Training the identification system on an air-written dataset can help it become more resistant to these fluctuations and enhance its capacity to handle noisy input.

**Enhanced User Experience:** Accurate detection of air-written numbers can lead to a simpler and more natural user experience in applications where users interact with touchless interfaces or virtual worlds.

**Improved Accessibility:** Because it provides an alternate input method for letter or numeric identification, air-writing recognition may aid those with physical limitations or motor impairments.

## 4 Experimental Design, Materials and Methods

### 4.1 Proposed Algorithm/Strategy for Dataset Preparation

Firstly, we have designed a GUI in Python to collect the dataset using a standard video camera for English and Devanagari numerals. A technique using the generic camera with a single writer and an uncontrolled environment is used to develop a dataset of numeral recognition. The system collects the videos of fingertip movements and saves them in the database. Subsequently, to track fingertip movement, continual notice, and recognition of air writing numerals, fingertip detection techniques are presented without external devices or sensors. Air writing in the real world may be recognized using laptop video cameras or webcams, making it easily accessible to all people. Video capture necessitates head-mounted smart cameras or mixed reality headsets like Google Glass, Microsoft HoloLens, and Facebook Oculus, which are only sometimes available to all users.

As a result, we leverage video inputs from a typical laptop camera or a webcam for the air-writing program to make our system more ubiquitous.

The air writing detection system comprises two steps, the first of which is palm detection and the second of which is writing identification. Palm detection also incorporates fingertip detection and gesture recognition to regulate writing activities. Our system can recognize digits from 0 to 9 in Devanagari and English Script/language. The numbers are written with the index finger and then in one of the two defined languages.

**Fig. 1:**
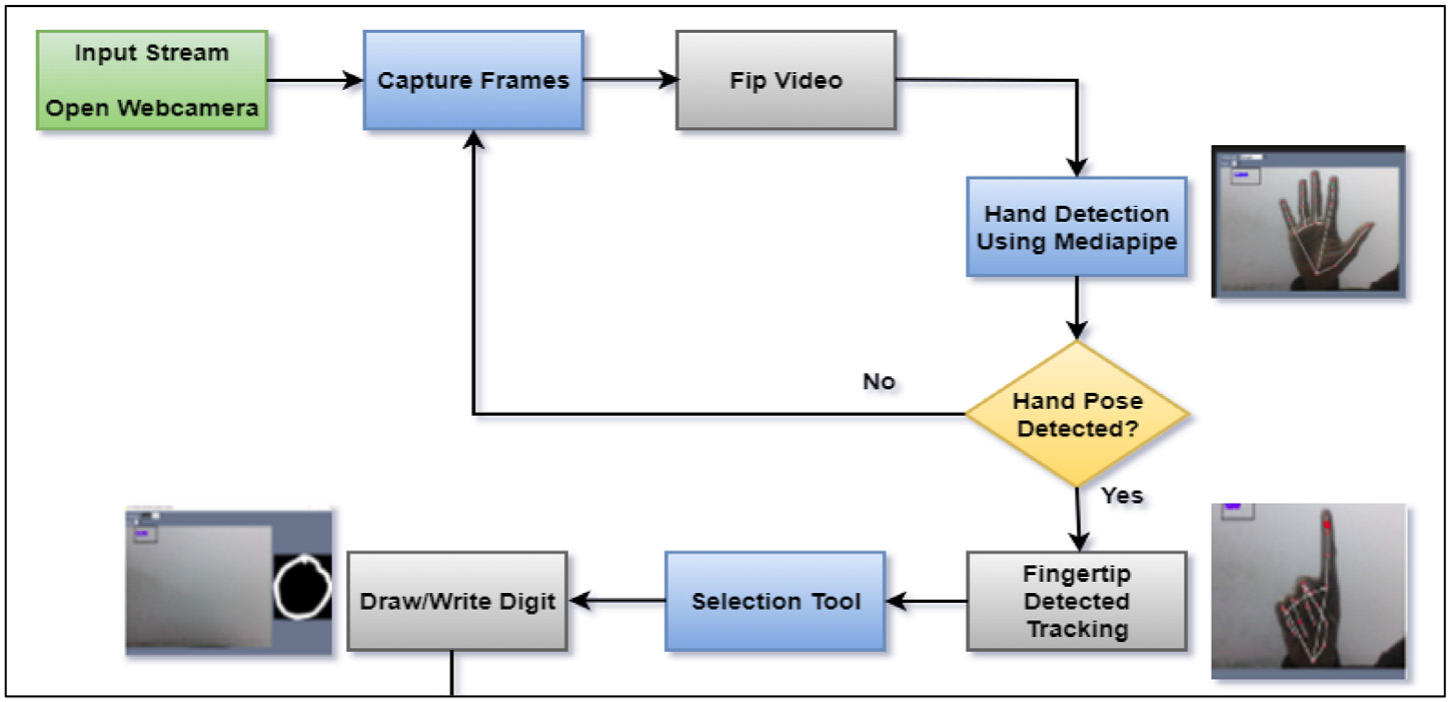
The proposed strategy for multilingual numeral dataset preparation.

Fig. 1 Depicts a flowchart for the suggested air-writing detection system. The proposed method is composed of the following four parts: (1) Video stream input and FIP input; (2) Palm and Hand pose detection; (3) Fingertip detection; writing hand pose detection and trajectory tracking; and (4) preprocessing and storing the number.

#### 4.1.1 Input Video Stream and Fip Video

In an air-written recognition system, we need to take the input from the webcam is an integral part of the system. We need to flip the video. Hence, the PySimpleGUI Python package has been used to create GUIs. We have provided the dropdown to select the language/script [3,4].

#### 4.1.2 Palm and Hand Pose Detection

Initializing air-writing requires detecting the writing hand posture and its differentiation from other movements. In contrast to traditional penmanship, which uses the pen-down and pen-up motions, air-writing lacks a clearly defined order of writing events. Five raised fingers are the definition of hand posture detection in this article, presuming the digit is unobstructed and not handling any objects. By numbering the number of raised digits, we can identify a right-writing hand and separate it from a non-writing hand posture. The current system is developed only for one hand in an uncontrolled environment. 21 hand landmarks were detected and composed with x, y, and z coordinates for width, height, and landmark depth, respectively [5].

**Fig. 2:**
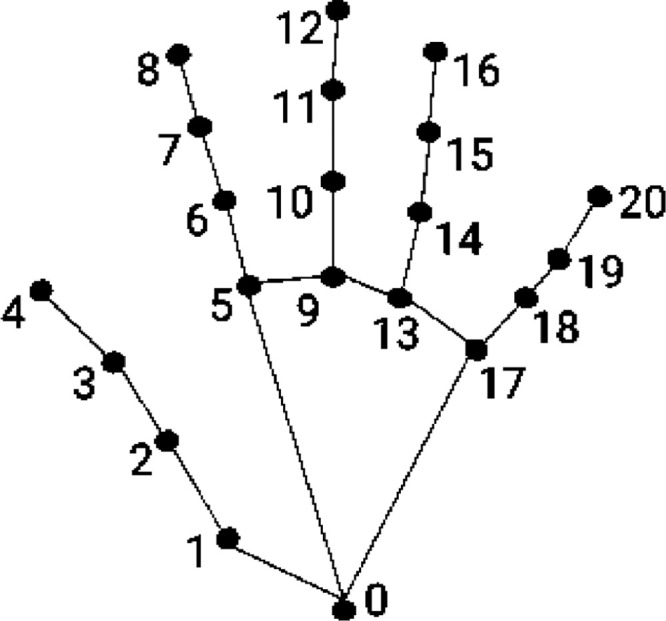
Hand tracking with 21 hand landmarks [4].

Subsequently, palm detection is followed by hand landmark detection [4]. Precise localization of hand landmarks is shown in Fig. 2, and a detailed description is given in Table 2. We used two-dimensional coordinates to learn real-world samples in the dataset and follow the landmark technique [6].

**Table 2:** Hand landmark and description [6].

Hand Landmark	Description	Hand Landmark	Description
0	Wrist	11	Middle Finger DIP
1	Thumb CMC	12	Middle Finger TIP
2	Thumb MCP	13	Ring Finger MCP
3	Thumb IP	14	Ring Finger PIP
4	Thumb TIP	15	Ring Finger DIP
5	Index Finger MCP	16	Ring Finger TIP
6	Index Finger PIP	17	Pinky MCP
7	Index Finger DIP	18	Pinky PIP
8	Index Finger TIP	19	Pinky DIP
9	Middle Finger MCP	20	Pinky TIP
10	Middle Finger PIP		

#### 4.1.3 Fingertip Detection, Writing Pose Detection and Trajectory Tracking

The fingertip detection is still challenging due to real-time video input and the small size of the fingertip. However, the hand landmarks define the fingertip with ease of hand segmentation. After hand segmentation, we must determine a fingertip position that can be differentiated with high curvature points and a considerable distance from the wrist landmark (0). These distinctive features simplify the accurate detection of fingertips. As shown in Table 2, points 4, 8, 12, 16 and 20 are localized as fingertips from the thumb to the pinky finger. However, our model initializes writing tasks using an index fingertip. Thus, different hand gestures are also defined to initialize and reset air writing [5].

We have defined hand gestures to control air writing activity using hand landmarks. It accepts the index fingertip as writing input, and other landmarks are not activated while writing in the video frame. Also, landmarks 1 and 9 are combined to initialize the writing by touching the thumb to the middle finger (Table 3). The digit drawn by the index fingertip (8) is saved by combining landmarks 4 and 13 on the thumb and ring finger, respectively. The frame can be cleared by closing all fingertips with a closed palm (Table 3) [7].

**Table 3:** Hand gesture details [5].

Sr. No.	Function	Hand Gesture	
		Landmarks	Fingers
1	Initialize	1, 9	Thumb and Middle finger
2	Save	4, 13	Thumb and Ring finger
3	Clear	No fingertips	Close all fingers

## Ethics Statements

The study does not involve experiments on humans or animals.

## CRediT authorship contribution statement

**Meenal K. Jabde:** Conceptualization, Data curation, Software, Visualization, Writing – original draft. **Chandrashekhar H. Patil:** Formal analysis, Funding acquisition, Project administration, Resources, Supervision. **Amol D. Vibhute:** Funding acquisition, Investigation, Methodology, Supervision, Validation, Writing – review & editing. **Shankar Mali:** Formal analysis.

## Data Availability

Meenal-Airwriting_Devanagari_English_numeral_dataset (Original data) (Mendeley Data)

## References

[1] Hsieh C., Lo Y., Chen J. (2021). Air-writing recognition based on deep convolutional neural networks. IEEE Acc..

[2] Ghosh T., Sen S., Obaidullah S.M., Santosh K.C., Roy K., Pal U. (2022). Advances in online handwritten recognition in the last decades. Comput. Sci. Rev..

[3] P. Roy, S. Ghosh, and U. Pal, “A CNN based framework for unistroke numeral recognition in air-writing,” no. August, 2018, doi:10.1109/ICFHR-2018.2018.00077.

[4] C. Lugaresi et al., “MediaPipe: a framework for building perception pipelines,” 2019, [Online]. Available: http://arxiv.org/abs/1906.08172.

[5] T. Watanabe, A. M. Hasan, H. Lee, and S. Jang, “2D Camera-based air-writing recognition using hand pose estimation and hybrid deep learning model,” pp. 1–14, 2023.

[6] Simon T., Joo H., Matthews I., Sheikh Y. (2017). Proceedings - 30th IEEE Conference on Computer Vision and Pattern Recognition, CVPR 2017.

[7] Mukherjee S., Ahmed S.A., Dogra D.P., Kar S., Roy P.P. (2019). Fingertip detection and tracking for recognition of air-writing in videos. Expert Syst. Appl..

